# Illuminating the Numbers: Integrating Mathematical Models to Optimize Photomedicine Dosimetry and Combination Therapies

**DOI:** 10.3389/fphy.2019.00046

**Published:** 2019-04-02

**Authors:** Bryan Q. Spring, Ryan T. Lang, Eric M. Kercher, Imran Rizvi, Robert M. Wenham, José R. Conejo-Garcia, Tayyaba Hasan, Robert A. Gatenby, Heiko Enderling

**Affiliations:** 1 Translational Biophotonics Cluster, Northeastern University, Boston, MA, United States,; 2 Department of Physics, Northeastern University, Boston, MA, United States,; 3 Department of Bioengineering, Northeastern University, Boston, MA, United States,; 4 Joint Department of Biomedical Engineering, University of North Carolina at Chapel Hill and North Carolina State University, Chapel Hill, NC, United States,; 5 Lineberger Comprehensive Cancer Center, School of Medicine, University of North Carolina at Chapel Hill, Chapel Hill, NC, United States,; 6 Department of Gynecologic Oncology, H. Lee Moffitt Cancer Center and Research Institute, Tampa, FL, United States,; 7 Department of Immunology, H. Lee Moffitt Cancer Center and Research Institute, Tampa, FL, United States,; 8 Wellman Center for Photomedicine, Massachusetts General Hospital and Harvard Medical School, Boston, MA, United States,; 9 Division of Health Sciences and Technology, Harvard University and Massachusetts Institute of Technology, Cambridge, MA, United States,; 10 Department of Diagnostic Imaging and Interventional Radiology, H. Lee Moffitt Cancer Center and Research Institute, Tampa, FL, United States,; 11 Department of Integrated Mathematical Oncology, H. Lee Moffitt Cancer Center and Research Institute, Tampa, FL, United States,; 12 Department of Radiation Oncology, H. Lee Moffitt Cancer Center and Research Institute, Tampa, FL, United States

**Keywords:** photomedicine, mathematical oncology, photodynamic therapy, immunotherapy, computational modeling, combination therapy, cancer

## Abstract

Cancer photomedicine offers unique mechanisms for inducing local tumor damage with the potential to stimulate local and systemic anti-tumor immunity. Optically-active nanomedicine offers these features as well as spatiotemporal control of tumor-focused drug release to realize synergistic combination therapies. Achieving quantitative dosimetry is a major challenge, and dosimetry is fundamental to photomedicine for personalizing and tailoring therapeutic regimens to specific patients and anatomical locations. The challenge of dosimetry is perhaps greater for photomedicine than many standard therapies given the complexity of light delivery and light-tissue interactions as well as the resulting photochemistry responsible for tumor damage and drug-release, in addition to the usual intricacies of therapeutic agent delivery. An emerging multidisciplinary approach in oncology utilizes mathematical and computational models to iteratively and quantitively analyze complex dosimetry, and biological response parameters. These models are parameterized by preclinical and clinical observations and then tested against previously unseen data. Such calibrated and validated models can be deployed to simulate treatment doses, protocols, and combinations that have not yet been experimentally or clinically evaluated and can provide testable optimal treatment outcomes in a practical workflow. Here, we foresee the utility of these computational approaches to guide adaptive therapy, and how mathematical models might be further developed and integrated as a novel methodology to guide precision photomedicine.

## INTRODUCTION

Photodynamic therapy (PDT) has been developed as a clinical therapy over the past century with origins dating back to ancient civilizations and applications for skin diseases using plant extracts and sunlight [1, 2]. Contemporary photodynamic chromophores for PDT, often termed photosensitizers (PS), feature enhanced photophysical properties for deep-tissue absorption of far-red laser light and for generating cytotoxic, activated chemical species (including reactive oxygen species). These modern PS are often engineered to rapidly clear from the skin and body to minimize the risk of severe sunburn—the major potential side-effect of otherwise non-toxic photomedicines [1, 2]. An example of clinical impact has been the use of PDT as a frontline therapy in ophthalmology for the wet form of age-related macular degeneration (wet AMD). PDT has benefited several 100,000 AMD patients worldwide since the early 2000s [3, 4]. In oncology applications, PDT o ers unique modes of cell death induction [5–7] that bypass many mechanisms of classical drug resistance [8], and tumor specificity with low toxicity to o -target tissues and organs when light activation is confined to the tumor site [1, 2]. A number of molecular-targeted PDT agents (e.g., antibody-PS conjugates) are emerging with enhanced tumor selectivity in preclinical models [9–16] as well as optically-active nanomedicine [17–19] to facilitate spatiotemporal control of multi-drug combination therapies that damage various tumor compartments in concert (e.g., cancer cells, tumor-supportive stromal cells, and microvasculature) while suppressing metastatic escape [19]. These advances promise to impact oncology more substantially than the present use of unformulated PS agents.

Another potential intrinsic advantage of photomedicine is that, rather than suppressing the immune system, it can stimulate acute inflammation, tumor-antigen exposure, and anti-tumor immunity [20]. A comprehensive review by Castano et al. summarizes the preclinical data demonstrating that PDT can be used to stimulate the host immune system [20]. For instance, it has been shown that pro-inflammatory cytokines are released following the administration of PDT in mouse models [20, 21]. In addition, PDT can increase the exposure of tumor-antigens to the host immune system. PDT-induced tumor necrosis facilitates the release of extra-cellular heat-shock protein (HSP) family members, such as HSP70 [20, 22]. HSP70 is a protein folding chaperone that stabilizes potential tumor-antigens and these HSP70–peptide tumor antigen complexes are thought to help stimulate an immune response through HSP binding to antigen-presenting cells, which in turn ultimately present the potential tumor antigens to CD8+ cytotoxic T cells [20]. However, PDT dosimetry is salient to maximizing anti-tumor immune stimulation vs. extensive tumor destruction that can counteract some of these mechanisms; e.g., high-dose PDT is more likely to interfere with immune cell tra cking in and out of the tumor by shutting down the tumor microvasculature. Gollnick et al. introduced an immune-enhancing PDT regime distinct from the conventional goal of maximal tumor damage [23]. Immune-enhancing PDT is performed at lower doses that promote acute inflammation and neutrophil infiltration into the tumor; and, a follow-up high-dose PDT regimen can be applied to reduce the tumor volume once the immune system is activated [23]. The use of low-dose and metronomic therapy is also being investigated for immune-enhancing effects for chemotherapy and radiotherapy [24, 25].

To the best of our knowledge, a comprehensive clinical study in humans of PDT-tumor immune modulation has not been performed although such a study is warranted. However, PDT-induced stimulation of the immune system is hypothesized as a candidate for clinical observations of increases in overall survival following PDT. A recent example is the finding that lung sparing surgery combined with intraoperative PDT of the chest cavity [26–28], followed by adjuvant chemotherapy, achieves remarkably extended survival for patients with malignant pleural mesothelioma compared to standard therapies [28]. The median overall survival of patients who received lung sparing surgery and intraoperative PDT is extended more than 2 years (3-year median overall survival) compared to patients treated by standard surgery with adjuvant chemotherapy (8-month median overall survival) [28]. The remarkable enhancement in survival extends ~2 years beyond the median time to disease recurrence (~1 year), which may suggest an anti-tumor immune-enhancing effect from PDT that also benefits from the sparing of lung tissue and the associated lymph nodes [28]. Inhibition of T cell egress from tumor-draining lymph nodes has recently been demonstrated to drastically limit antitumor immunity post radiation [29], spurring discussion for investigations into lymph node sparing in radiotherapy [30].

Like radiation therapy, PDT requires robust production of activated chemical species in order to illicit inflammation and cell death. PDT-based treatments involve the administration of a PS, which is non-toxic until it absorbs visible light, and the PS then facilitates the conversion of molecular oxygen into reactive oxygen species, among other photochemical mechanisms of producing activated reactive species, which are known to damage tumors by several mechanisms [2]. For instance, PDT can be applied to curtail the tumor microvasculature to starve tumors of nutrients [31], and it is also possible to produce direct tumor cell death via apoptosis, necrosis and other cell death pathways [7, 32], or a combination of tumor vascular and cellular effects [33]. Importantly, the PS-light interval can impact which compartment of the tumor receives the damage based on the pharmacokinetics of the PS [33]; and, the subcellular localization of the PS also determines the stimulation of specific cell death pathways and the interaction of PDT with other modes of therapy [7, 8, 34–36].

Given the intricacies of PDT dosimetry, an ongoing dilemma is the lack of robust PDT dosimetry tools. These technologies are still maturing, and cutting-edge advances have yet to be translated to the clinic. However, advanced imaging modalities report prognostic indicators of biological responses [37], and significant advances in developing both explicit and implicit metrics of the photodynamic dose deposited in target sites have yielded exciting data ripe for rigorous analysis [37–41]. However, very little attention is given in general to *a priori* optimization of dose, dose fractionation, treatment timing, and sequencing of synergistic treatments among oncologists and cancer researchers. An exhaustive preclinical and clinical evaluation of all possible doses and treatment combinations is impossible. Recently, progress in integrated mathematical oncology, a powerful approach that iteratively utilizes experimental and clinical data to build calibrated quantitative models to predict response to untested treatments and treatment combinations, makes such complex analysis approachable [42, 43]. Development, calibration, and validation of quantitative and predictive models are critical in order to focus experimental and clinical trial design on regimens that maximize efficacy and quality of life.

## INTEGRATED MATHEMATICAL ONCOLOGY

Mathematical modeling in cancer research has a long history [42–47]. Mathematical oncology approaches often incorporate first-order mechanistic principles of tumor growth laws [48, 49], ecology, and evolution [50–53], and increasingly interactions with the immune system [54–59] to simulate biological responses to a variety of therapies [60–66]. One class of mathematical models may follow a “bottom-up” approach, where mechanisms on specific biological or temporal scales (i.e., subcellular, or cellular dynamics) are described mathematically, and simulations reveal emergent properties on larger scales (cellular, tissue level, organ level). Vis-à-vis “bottom-up” is the top-down approach, where modeling the dynamics on a higher level (for example a tumor population) may provide insights on the underlying mechanisms of the system (for example, cellular properties, and cell-cell interactions). A detailed summary of the most appropriate mathematical techniques for the Different biological, temporal, and spatial scales with illustrative examples can be found elsewhere [67].

One recent application of successful mathematical oncology has demonstrated the translation of the ecological and evolutionary principles of emerging cancer treatment resistance into a clinical trial of adaptive therapy of metastatic castrate-resistant prostate cancer [68]. Simulations of a simple evolutionary game of three competing cancer cell subtypes and their distinct susceptibilities to a single drug (abiraterone, an inhibitor of testosterone production used for androgen-deprivation therapy) suggest that, instead of aiming for an impossible cure with continuous treatment, adaptive intermittent therapy could control the tumor by maintaining competition among the three cancer cell subtypes. With this approach, the androgen-independent cell population is not able to proliferate and establish the treatment refractory, and ultimately lethal, abiraterone-resistant disease [68]. In the prospective clinical study of adaptive hormone treatment, the majority of patients maintained stable oscillations of tumor burden for at least 27 months (10 of 11 patients were still progression free with stable tumor burden at the time of publication) using only 47% of the normal cumulative drug dose, whereas the median time to progression is 16.5 months for standard dosing [68]. An alternative strategy to successfully combat resistance may be to not target individual cell populations, but to change the fitness and cost of their interactions with each other and their tissue environments [69].

A wealth of knowledge exists already in the literature regarding the intricacies of PS pharmacokinetics, PS cellular localization, and photodynamic dosimetry [2]; and, there is considerable preclinical data deciphering the complex spatiotemporal interplay of PDT with the tumor and the tumor microenvironment that may increase the likelihood of treatment success [2]. Many of the biological factors influencing the efficacy of PDT as well as molecular mechanisms of tumor response to PDT have been elucidated. While clinical trials for new therapies are plentiful (including PDT), with a few exceptions, most are based on limited pre-clinical studies with arbitrarily chosen treatment parameters. This motivates the development and application of mathematical and computational models that capture salient features from the preclinical and clinical knowledgebase to make reliable predictions by conducting the experiments *in silico* to identify a small set of potentially optimal protocols for experimental testing. Unexpected novel insights and breakthroughs may also be garnered from the simulations that are hard to anticipate due to the multiparameter nature of combination therapies and tumor biology—such as the hypothesis that adaptive therapy with intermittent dosing and drug holidays [53, 68, 70–76] can lead to stable tumor oscillations that has now shown remarkable promise in the clinic [68].

**FIGURE 1 |:**
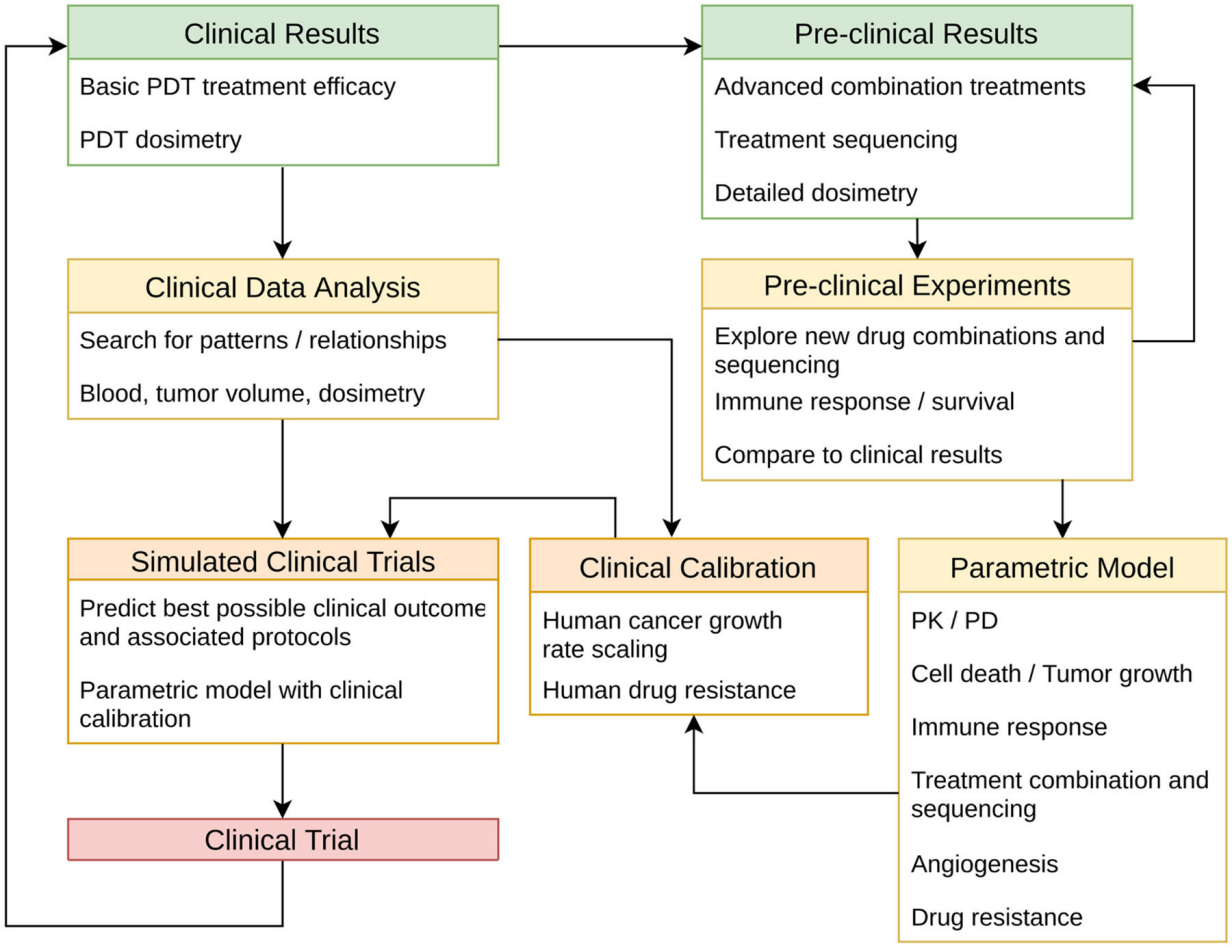
Proposed work flow for integrating pre-clinical and clinical data with quantitative models of PDT dosimetry, synergies with other therapies, and interactions with tumor biology. The model is calibrated to unify observations from human data and preclinical biological models, and applied to simulate many possible clinical treatment regimens. Optimal regimens are then identified and can be tested in preclinical and clinical studies. Further, results from biological models and human studies may then be used to further refine the computational model as needed in an iterative approach. Online simulation may also be used to guide personalized and adaptive therapy tailored to individual patients and tumor biology dynamics using feedback from clinical tests and imaging data throughout the course of the treatment. Color-coding suggests time-to-realization for these steps: green boxes indicate that a great deal of data is available in the existing literature; yellow boxes indicate experiments and models that can be immediately developed; orange boxes indicate steps that require preliminary work to be done first; and, the red box (Clinical Trial) represents the overarching goal for this workflow. PK, pharmacokinetics; PD, pharmacodynamics. Adapted from McGuire et al. [60].

The fundamental importance of photodynamic dosimetry for achieving local tumor control, drug-release, and synergistic interactions with other therapeutic modalities, has motivated the development of mathematic models of photochemistry and photophysics as well as dosimetry and dose deposition [39, 77–80]. Other modeling studies focused on identifying optimal oxygenation conditions for PDT effects [81, 82], and on information processing and cellular decision-making during PDT to predict cell survival [83]. In a number of recent studies, the effect of cell death through apoptosis and necrosis was quantitatively modeled [84, 85]. These studies suggest that PDT is most likely successful in tumors with high surface-to-volume ratios, and that PDT is unlikely to provide control in fast proliferating deep tumor tissues, which supports previous model results for low-penetrating red-light PDT [86]. As PDT and radiation therapy share similar biological responses and routine involvement of medical physics, modeling approaches for radiotherapy response may be readily translatable to identify optimal PDT protocols [59, 61, 63, 87–93]. In this Perspective, we suggest integrated mathematical oncology as a computational platform for developing quantitative models and simulations of PDT dosimetry to optimize local tumor control, tumor-focused drug release, spatiotemporal dynamics, and photodynamic priming of systemic modes of therapy. Based on the wealth of available mathematical models and increasingly emerging data on PDT, it is conceivable that established mathematical oncology workflows can be adapted to PDT. Integrating preclinical and clinical experience with computational models and simulation, in an iterative approach with continuous refinements based on observed results, will identify the most promising protocols for subsequent experimental and clinical validation (Figure 1). This computational model-guided approach to research and clinical oncology promises an efficient, cost-effective, and safe approach to synergize with experimental study design.

## TOWARD A MATHEMATICAL MODEL OF IMMUNE-ENHANCING PDT

Cancer therapy success may be a combination of the direct lethal effect on cancer cells and, possibly more importantly, the subsequent indirect effect of stimulating a potent antitumor immune response. Tumors grow in a complex ecosystem that is the result of co-evolution of the tumor with its host environment. A contribution of the complex tumor microenvironment to treatment outcome is increasingly appreciated. Focal therapies like PDT and radiation can potentially induce a robust antitumor immune response that provides a second wave of cell kill and tumor regression [94, 95]. Functional immunity is comprised of two main conceptual components: (1) immune effector populations that act to regress the tumor including natural killer (NK) cells, N1 neutrophils, CD4+ helper T (Th) cells, CD8+ cytotoxic (CTL) T cells, M1 macrophages and mature dendritic cells (DC); and (2), immune suppressor cells that facilitate tumor escape, including N2 neutrophils, regulatory T (Treg) cells, myeloid-derived suppressor cells (MDSC), M2 macrophages, and tolerogenic DC [96]. After immune effector populations become activated against a pathogen or, as in cancer, against cells presenting abnormal antigens, immune suppression is a natural response to prevent autoimmune diseases. As an example, Tregs can suppress antitumor immunity through a variety of mechanisms, including inhibition of DC maturation and function, release of inhibitory cytokines such as TGFβ, and high expression of the IL-2 co-receptor CD25 that deprives the environment of IL-2 and thereby disrupts CD8+ T cell proliferation and granzyme A and B-dependent effector T-cell cytolysis [97]. Our present understanding of this network of immune effectors vs. immune suppressors can in principle be modeled to guide the development of therapies that can help shift the local tumor environment away from immunosuppression by bypassing or reducing checkpoints to effector immune cell activity.

Despite recent advancements in cancer immunotherapy, mainstay cancer therapies have not yet specifically focused on enhancing the immune response to tumor antigens to help eliminate or control tumors. Radiation therapy, for example, increases the mutational burden and induces cell stress as well as immunogenic cell death, thereby exposing an array of otherwise hidden and *de novo* tumor-associated antigens, stress proteins, and danger-associated molecular patterns to the immune system [98, 99]. Yet standard daily radiation over many weeks may be detrimental to antitumor immunity, as CD8+ T cells are generally very radiation sensitive [100]. Tumor infiltrating lymphocyte (TIL) enrichment after neoadjuvant radiation (RT before surgery) was previously assessed for 40 rectal cancer patients. The densities of CD3+ and CD8+ T lymphocytes significantly increased from pre-treatment biopsy specimens to post-treatment surgically resected specimens [101]. Neoadjuvant RT for early stage breast cancer significantly improves disease-free survival compared to radiation after surgery, arguably due to induction of robust antitumor immunity and immune memory [102]. Numerous clinical trials are currently underway exploring Different radiation dose and dose fractionation protocols for clinical efficacy against the targeted tumor and induction of systemic antitumor immunity. However, the large number of possible treatment protocols make a trial-and-error approach unlikely to be successful, and the quest for optimal treatment protocols and treatment combinations may need to include mathematical modeling [30]. As recently demonstrated for radiation-induced local and systemic antitumor immunity, mathematical models may be parameterized by preclinical and clinical data to simulate an array of possible treatment regimens with varying therapeutic modalities, drug dosages, and treatment sequences [44]. Model predictions of optimal treatments can be tested in pre-clinical studies and ultimately prospective clinical trials.

Enhanced selectivity of PDT presents a great opportunity to overcome the limitations of chemotherapy and radiotherapy to potentially impact the efficacy of immune checkpoint inhibitors, and guidance from mathematical modeling may be readilyavailable. The numerous models on tumor-immune interactions studies [55–57, 103] and radiation response modeling studies [59, 89, 104–106] may translate directly to PDT, albeit with Different model parameters. These can be obtained from relatively simple and well thought-out *in vitro* and in vivo studies (Figure 1).

## CONCLUSIONS

In summary, PDT o ers unique selectivity and mechanisms of action for eliciting tumor damage, overcoming classical drug-resistance, and enhancing anti-tumor immunity with a toxicity profile distinct from standard radiation and chemotherapy. To fully harness the benefits of PDT requires complex dosing and scheduling with concurrent treatments. Even arguably simple mathematical models can provide deep insights, and we forecast that the power and utility of integrated models will grow as the molecular underpinnings and multi-scale behavior of the complex tumor ecosystems is further elucidated and incorporated into these models. The rapid advancement of bioinformatics to characterize tumor genetic, transcriptome, and epigenetic heterogeneity as well as molecular mechanisms of interactions with the host microenvironment and immune system will greatly advance current phenomenological models—with the ultimate goal to create a multi-scale computational platform that integrates molecular-level detail, evolutionary principles, and systems biology.
